# Promoting Resilience among Middle-Aged and Older Men Who Have Sex with Men Living with HIV/AIDS in Southern Nevada: An Examination of Facilitators and Challenges from a Social Determinants of Health Perspective

**DOI:** 10.3390/healthcare11202730

**Published:** 2023-10-13

**Authors:** Brandon Ranuschio, Sherry Bell, John M. Waldron, Lianne Barnes, Nadia Sheik-Yosef, Esmeralda Villalobos, Janelle Wackens, Renato M. Liboro

**Affiliations:** 1 Department of Psychology, College of Liberal Arts, University of Nevada, Las Vegas, NV 89154, USA; fraga@unlv.nevada.edu (B.R.); bells12@unlv.nevada.edu (S.B.); lianne.barnes@unlv.edu (L.B.); sheikn1@unlv.nevada.edu (N.S.-Y.); villae12@unlv.nevada.edu (E.V.); wackens@unlv.nevada.edu (J.W.); 2 LGBTQIA+ Community Center of Southern Nevada (The Center), Las Vegas, NV 89101, USA; jwaldron@thecenterlv.org; 3 Centre for Addiction and Mental Health, Toronto, ON M5S 2S1, Canada

**Keywords:** resilience to HIV/AIDS, middle-aged and older, men who have sex with men, social determinants of health, facilitators and challenges

## Abstract

Most prior research on resilience to HIV/AIDS among middle-aged and older men who have sex with men (MSM) has utilized quantitative methods that employ surveys and scales to measure constructs researchers have used to approximate the concept of resilience to HIV/AIDS. Only a few studies have purposively made efforts to incorporate the input of relevant stakeholders to guide their research on HIV/AIDS resilience and examine the perspectives and lived experiences of middle-aged and older MSM. To address this research gap, we conducted a community-based participatory research qualitative study to examine the perspectives and lived experiences of HIV-positive, middle-aged and older MSM from Southern Nevada in order to identify factors that promote such resilience. We conducted 16 semi-structured interviews with middle-aged and older MSM living with HIV/AIDS from January to April 2022. From our thematic analysis of our interviews, we identified factors that served as facilitators or challenges to the promotion of our participants’ HIV/AIDS resilience. We discuss in this article both the facilitators and challenges to our participants’ resilience-building as the key themes from our interviews. We recognized that the impacts of these factors were mediated by their strong influence on the social determinants of health that were explicitly relevant to our participants. We offer important insights based on our findings, which could be especially useful to future research on resilience to HIV/AIDS.

## 1. Introduction

Resilience is the process and outcome of successfully adapting to difficult or challenging life experiences, especially through mental, emotional, and behavioral flexibility and adjustment to external and internal demands [1]. It has been described in the academic literature as a multi-system process between the self, others, and the surrounding community [2], and has been reported not only to be of great value to bolstering mental health, but also protective against the effects of trauma and stigma, often acting as a buffer against various types of stressors [3,4,5]. Resilience has been defined as a positive adaptation within the context of significant adversity [6], and has been commonly referred to as the ability to recover from, withstand, or overcome significant stress or illness [7], including chronic forms of illness such as cancer, autoimmune diseases, and HIV/AIDS [8,9,10,11,12,13].

In research exploring the value of resilience to HIV/AIDS, scholars have examined the essential role of resilience in meeting ongoing and evolving global HIV goals, drawing attention to personal conceptualizations of resilience among people living with HIV/AIDS (PLWH) and the delineation of factors that PLWH identify as critical to promoting their ability to cope with HIV-related challenges [11]. The scientific study of resilience among HIV/AIDS populations has continued to grow in the 21st century [11,14,15]. Researchers focused on strengths-based approaches for developing interventions to address various issues related to HIV/AIDS have increasingly recognized the important role of resilience in the focus of their scholarly work [16,17].

Among the different groups affected by HIV/AIDS, ethnoracially diverse, middle-aged and older men who have sex with men (MSM) living with HIV/AIDS have been the specific subpopulation that has been impacted by HIV/AIDS the longest [18,19], and likely the group that has exhibited resilience to HIV/AIDS the most since the beginning of the epidemic [20]. Evidence of resilience to HIV/AIDS among middle-aged and older MSM has been documented and discussed in research articles [21,22,23], and consequently, resilience to HIV/AIDS has been reported to be key to the successful aging of MSM living with HIV/AIDS [24]. In their quantitative, descriptive, comparative, and cross-sectional study, Batista and Pereira reported HIV-positive older gay, bisexual, and other MSM to have higher than expected levels of resilience [25]. This is potentially because, along with other older PLWH, middle-aged and older MSM have been reported to be able to build their levels of resilience by positively adapting to different types of adversity [26]. When utilizing a strengths-based approach in research involving older MSM living with HIV/AIDS and other PLWH, an emphasis on resilience not only helps ensure that the research process does not primarily focus on pathology, weaknesses, and risks, but also encourages research efforts to empower individuals by focusing their attention on controllable aspects of life [27].

Recent studies have explored and discussed a variety of factors that have either prospectively served as facilitators to the promotion of resilience to HIV/AIDS among MSM living with HIV/AIDS, or as barriers and challenges to such resilience-building [20,28,29,30,31]. Examples of facilitators that have been explored include individual characteristics such as perseverance [30,31]; external protective factors such as social support from family and friends, religion and spirituality, and education [16,20,26]; and community-level resources such as lesbian, gay, bisexual, trans, queer, and intersex (LGBTQI+) not-for-profit agencies and AIDS services organizations [17,32,33,34]. Examples of challenges that have been examined in the research include HIV stigma and discrimination (i.e., negative attitudes toward HIV/AIDS and the negative treatment of PLWH that have been found to exacerbate social isolation and other co-occurring psychosocial health problems) [17,20,24,30,31,35], cultural and language barriers, and racism [30]. Other studies have discussed factors that may have served as facilitators or challenges to promoting resilience to HIV/AIDS among middle-aged and older MSM living with HIV/AIDS and other PLWH, and described how these factors may have exerted their influence through their impacts on various social determinants of health [36,37].

### 1.1. A Social Determinants of Health Perspective

Social determinants of health are the non-medical factors in society that influence health outcomes, and are the conditions in which people are born, grow, work, live, and age, as well as the wider set of forces and systems shaping the conditions of daily life [36,37]. They have an important influence on health inequities, which are avoidable inequalities in health between groups of people within countries and between countries. Socioeconomic conditions and their impacts on people’s lives determine their risk of illness and the actions taken to prevent them from becoming ill or treat their illness when it occurs [36]. The World Health Organization (WHO) enumerates and describes a list of social determinants of health, which can influence health equity in positive and negative ways. This list includes, but is not limited to: (1) education; (2) income and employment; (3) access to quality health services; (4) food security; (5) housing stability; and (6) social inclusion and non-discrimination [37]. Social determinants of health present as an important framework, and prospective scholars could consider them when examining health disparities that affect the wellbeing of underserved populations and people at the margins, including middle-aged and older MSM living with HIV/AIDS. In this article, we examine the findings of our study through a social determinants of health perspective, particularly from the specific framework described by the WHO [37].

### 1.2. Research on Resilience to HIV/AIDS among Middle-Aged and Older MSM and Our Study

It is very important to note that most prior research that has been conducted to explicitly focus on and examine resilience to HIV/AIDS, specifically among middle-aged and older MSM, has utilized quantitative and statistical methods that employ surveys and scales to measure constructs that researchers themselves have identified, chosen, and used to approximate the concept of resilience to HIV/AIDS [3,22,25,38]. To the best of our knowledge, in the past decade, only a few scholars have purposively made efforts to not only include the direct input of relevant community stakeholders to define the concept of resilience to HIV/AIDS and guide their research, but also examine the perspectives and lived experiences of middle-aged and older MSM, so as to gain a more in-depth understanding of factors that impact their resilience to HIV/AIDS [17]. Based on our literature search for studies that have both deliberately involved the input of relevant community stakeholders to explicitly define and describe their resilience to HIV/AIDS, as well as examined the perspectives and lived experiences of middle-aged and older MSM living with HIV/AIDS through in-depth, confidential interviews, we were only able to identify a few pertinent studies, all of which were conducted, and at this time, regionally defined, within the context of the Greater Toronto Area and the central and southwestern regions of Ontario, Canada [20,30,31]. Most importantly, none of these studies have examined factors that promote resilience to HIV/AIDS from a social determinants of health perspective. To address these different research gaps, our community-based participatory research (CBPR) qualitative study, described in this article, examined factors that have impacted the promotion of resilience to HIV/AIDS among middle-aged and older MSM living with HIV/AIDS in Southern Nevada from a social determinants of health perspective [36,37].

We conducted our CBPR qualitative study in Las Vegas and the greater area of Southern Nevada, which is a sprawling region that has many features and characteristics distinct from other large urban cities of North America [39,40,41,42,43,44,45,46]. For the purposes of our study, we operationalized the definition of resilience to HIV/AIDS in close consultation and collaboration with our community partners as the capacity of middle-aged and older MSM living with HIV/AIDS to: (1) survive the clinical and social impacts of living with HIV/AIDS, (2) live full lives despite having a chronic illness, (3) thrive despite challenges brought about by HIV-related discrimination, and/or (4) purposefully contribute to the goal of ending the HIV/AIDS epidemic.

## 2. Materials and Methods

### 2.1. Partnerships and Collaborations

We conducted the qualitative study discussed in this article as part of a larger, mixed-methods, CBPR project, in collaboration with our primary community partner, The LGBTQIA+ Community Center of Southern Nevada (The Center). The Center was instrumental in connecting us with a larger network of community-based agencies, AIDS service organizations, clinics and community health centers, and other relevant stakeholder groups from Southern Nevada, which were all dedicated to providing health and social services specifically to PLWH in our region, including middle-aged and older MSM living with HIV/AIDS. The Center also played an important role in creating a Community Advisory Board (CAB) composed of healthcare and service providers from other relevant Southern Nevada agencies such as the Golden Rainbow, Southern Nevada Health District, and Community Counseling Center of Southern Nevada who were involved in our project from the very beginning, collaborating with our research team on several research process stages such as finalizing our research focus, designing our research method, recruiting our study participants, evaluating and ratifying our findings from our data analysis, and disseminating our study findings and lessons learned to the rest of the Southern Nevada community. Our CAB members provided us with timely and valuable input and feedback during the research process, which helped our research team respond and adjust accordingly to both anticipated and unexpected issues such as matters related to participant recruitment and occasional miscommunication.

### 2.2. Participants

Prior to conducting our study, we obtained ethics approval for our research protocol from the Institutional Review Board (IRB) of the University of Nevada, Las Vegas (IRB protocol #1657449-2). During our participant recruitment process, we utilized IRB-approved printed flyers that we posted on the premises of our community partner agencies and organizations, and recruitment messages that we made accessible through our community partners’ various email listservs and websites. We became actively involved in several community events that our community partners sponsored and organized, which allowed us to personally and directly hand out IRB-approved recruitment flyers and pamphlets to prospective participants during these events. Working with numerous community partners, we recruited participants from Southern Nevada, all of whom participated in our semi-structured interviews. We continued to recruit and interview participants until data saturation for key themes was achieved (i.e., no new information relevant to the key themes emerged as additional interviews were conducted). In order to qualify, participants needed to (1) be 40 years of age or older, (2) have lived with HIV/AIDS for at least 1 year, (3) currently reside in Southern Nevada, and (4) be someone who self-identified as gay, bisexual, queer, or a man who has sex with men. Our inclusion criteria ensured that our participants (*n* = 16) were middle-aged and older MSM living with HIV/AIDS from Southern Nevada, and had enough lived experience with HIV/AIDS to develop and form their own perspectives and insights. Our participants’ ages ranged from 41 to 68 years old, with a mean age of 54. All 16 participants self-identified as gay and were regularly taking prescribed antiretroviral therapy medications at the time of their study participation. In terms of race, our participants identified as White (50%, *n* = 8), Black (37.5%, *n* = 6), Asian-Pacific Islander (6.25%, *n* = 1), and Middle Eastern (6.25%, *n* = 1). We assigned each of our participants a pseudonym when they joined our study, and we subsequently used their respective pseudonyms to identify them in this article (see Table 1 for all participant demographics). Each participant provided express written consent to join our study, and received a USD 50 gift card at the end of their participation as compensation for their time and efforts.

### 2.3. Procedures and Materials

Adhering to our IRB-approved research protocol, we conducted our confidential, one-on-one, semi-structured interviews remotely over Zoom between January and April of 2022, and on average, our interviews lasted from 40 to 60 min. Our interviews addressed questions that were deemed important both by the findings of the recent quantitative study we conducted as part of the larger, mixed-methods CBPR project we based in Southern Nevada [47], and by our community partners. Based on the preliminary results of the quantitative study we conducted in Southern Nevada, we were able to document that (1) knowledge on HIV/AIDS and (2) family support were significant predictors of resilience to HIV/AIDS [47]. Our previous study analyzed online survey responses that assessed for individuals’ level of resilience to HIV/AIDS, previous knowledge on HIV/AIDS, and family support. Our preliminary results demonstrated a negative correlation between knowledge on HIV/AIDS and resilience to HIV/AIDS, in which higher scores from correctly answering questions regarding HIV/AIDS (e.g., Can one become HIV infected by donating blood?) were related to lower levels of resilience to HIV/AIDS (*r*(33) = –0.36, *p* = 0.034). Contrastingly, we found a positive correlation between family support and resilience to HIV/AIDS, in which higher levels of support received from family were related to higher levels of resilience to HIV/AIDS (*r*(33) = 0.37, *p* = 0.026). In order to obtain valuable input from our community partners, we shared these results with them prior to creating a community report for dissemination to the larger community, and solicited their feedback. Not only did our community partners find our survey results interesting, but they also deemed it important for our research partnership to further explore the possible rationales and implications of our quantitative findings through one-on-one interviews with our participants who expressed interest in joining the qualitative stage of our CBPR project during the time they participated in our survey.

Together, we customized our interview guide questions so that we could explore how and where our participants gained their knowledge on HIV/AIDS, and why an increase in their knowledge on HIV/AIDS could be related to lower resilience to HIV/AIDS, as well as better understand what family support actually meant to them, and how family support was able to help them build their resilience. We tailored our interview guide questions so that they would be able to address our operational definition of resilience to HIV/AIDS. We included questions in our interview guide that would help us explore our participants’ perspectives and lived experiences, and more specifically, understand factors they believed helped them survive the clinical and social impacts of living with HIV/AIDS, live full lives despite having a chronic illness, thrive despite the challenges they encountered as middle-aged and older MSM living with HIV/AIDS in Southern Nevada, and purposefully contribute to the goal of ending the HIV/AIDS epidemic. For instance, we asked our participants open-ended questions in order to encourage them to spontaneously elaborate on their own experiences. These included general questions such as, “How do you think you were able to persevere in the face of health and social challenges brought about by HIV/AIDS?”, as well as more pointed prompt questions such as, “What do you think has helped lower your health risks in relation to HIV/AIDS over the years?”, and “What kind of resources are available to you that you believe help you access essential HIV-related care and services?”. We recorded our interviews with each participant’s consent, and later, de-identified and transcribed them verbatim for our analysis.

### 2.4. Analysis of Data

We analyzed our de-identified transcripts using the thematic analysis phases previously described by Braun and Clarke [48]. Due to its inherent flexibility, we chose thematic analysis as our method to analyze our interview data. We deemed it as the best approach to fulfill our study’s goals because its epistemological and theoretical freedom allowed for a flexible examination of the different perspectives we derived from our participants [48]. Braun and Clarke’s thematic analysis method is an iterative process that consists of six phases: (1) becoming familiar with the data, (2) generating codes, (3) generating themes, (4) reviewing themes, (5) defining and naming themes, and (6) locating exemplars [48]. To execute the first phase of our analysis, we used the first half of our data set of 16 interviews to develop our initial thematic codebook. Reviewing the first eight transcripts of our study’s data set provided the more seasoned coders of our research team a considerable opportunity to familiarize themselves with our interview data. Our research project coordinator and two of this article’s authors separately read and re-read our first eight transcripts to thoroughly familiarize themselves with the interview data, and then subsequently gathered together to discuss potential common codes and themes during bi-weekly meetings as initial coders. Upon reaching a consensus based on the review and coding of our first eight interviews, our initial coders finalized our codebook with key themes and sub-themes to execute the second to fifth phases of our thematic analysis [48]. Our initial coders then shared the codebook with three other members of our research team, who served as additional coders. All six coders proceeded with analyzing our remaining eight transcripts using the finalized codebook as a guide during the remainder of our analytic process. For the final phase of our analysis, all six coders continued with bi-weekly meetings throughout the analytic process until an agreement was reached on our interview data’s key themes, sub-themes, and representative codes and quotes.

## 3. Results

After completing the thematic analysis of our 16 interviews, we identified two key themes and four sub-themes from our interview data, all within the specific context of Southern Nevada. The two key themes we identified represented: (1) facilitators to promoting resilience to HIV/AIDS, and (2) challenges to promoting resilience to HIV/AIDS. Under the key theme, facilitators to promoting resilience to HIV/AIDS, we identified two sub-themes: (1) a strong network of local HIV/AIDS prevention, treatment, and related social services, and (2) emotional and mental health support from family of origin or chosen family. Under the key theme, challenges to promoting resilience to HIV/AIDS, we also identified two sub-themes: (1) the absence of a central hub for HIV/AIDS care and services, and (2) persistent HIV stigma. The qualitative findings from the analysis of our interview data that could potentially explain our quantitative results regarding the negative correlation between knowledge on HIV/AIDS and resilience to HIV/AIDS are beyond the scope of this article and will be discussed elsewhere in a future research article.

### 3.1. Facilitators to Promoting Resilience to HIV/AIDS

As our participants candidly discussed, in their interviews, their perspectives and insights on factors related to the promotion of their resilience to HIV/AIDS based on their lived experiences as middle-aged and older MSM living with HIV/AIDS, certain factors stood out as important facilitators to their resilience-building over the years. These facilitators were able to promote their resilience specifically by helping them address both their physical health needs, as well as their emotional and mental health needs.

#### 3.1.1. A Strong Network of Local HIV/AIDS Prevention, Treatment, and Related Social Services

As residents of Southern Nevada, most of our participants described having a strong network of local HIV/AIDS prevention, treatment, and related social services in our region as a crucial facilitator to promoting their resilience to HIV/AIDS. This strong network of services our participants described is composed of over half a dozen clinics and community health centers that have provided specific health care to PLWH, as well as numerous interconnected, community-based, not-for-profit agencies and AIDS service organizations, which have not only linked our participants to critical health-related (e.g., sexual health, mental health, and counseling) services, but just as importantly, introduced them to practical social support programs and services (e.g., food banks, housing subsidy and vouchers, insurance coverage, and employment assistance) to help meet their most basic needs, all within their network. Many participants emphasized how important it was for them to receive such practical social support because it allowed them to focus their efforts on staying actively engaged in the HIV/AIDS care continuum without having to excessively worry about day-to-day concerns such as where they would get their next meal or having a roof over their heads. Roy (41 years old, White, HIV-positive for seven years) explained:

Of course, with funding from [an AIDS service organization], not only do I get better access to care with providers who really know their stuff about LGBTQI+ folks and HIV, but their program has also helped me out with dealing with my food insecurity, you know. So, there’s some absolutely amazing resources here in Las Vegas to help us keep going.

Many participants reported that they needed to make use of food banks and other types of dietary aid, which they were able to access thanks to the referrals they obtained from the local community-based agencies within the strong network of services they described. Some participants even shared how much they appreciated the assistance they received from clinic or agency nutritionists who helped them establish better dietary goals and eating habits to stay healthy. Participants described how during their clinic consults with their healthcare providers, they would get referrals to local AIDS service organizations, which in turn connected them to other agencies with healthy dietary programs. Roy (41 years old, White, HIV-positive for seven years) described the eventual end result from the series of referrals he received within the strong network:

One grant program, the one run by [a local agency], was able to connect me to an [LGBTQI-affirming] nutritionist who has really helped me learn about better eating habits. Like I said, when I mentioned my food insecurity earlier, they were able to get grant money to buy me a box of groceries once a month for four months, while I was implementing my new nutrition regimen. The program was a lot of help!

Other participants expressed their gratitude for the admirable work ethic of those working or volunteering in the network’s local organizations, particularly those from organizations that helped them meet eligibility requirements for obtaining health insurance, housing support, and financial assistance. Many participants noted how most of their healthcare and service providers knew each other and worked well together within their local network, and consequently expressed how much they appreciated some providers for their passion and willingness to help by going above and beyond their work responsibilities. Peter (50 years old, Middle Eastern descent, HIV-positive for 29 years) described the providers he admired within their strong and growing local network:

And they’re really, really nice people. I mean, they really work hard. They work long hours to raise money and get us benefits. I believe they’re in the same boat too, so that makes them want to [fundraise] well even more. Something happened to my insurance once, a long time ago. I went to them for help and they didn’t even ask any questions. They just took care of it. So there’s more and more places that can help. You just have to get the right connections.

Our participants found dependable sources of emotional and mental health support from the community organizations of their region’s strong network of services. Hugh (56 years old, Black, HIV-positive for 22 years) underscored the personal importance of the support group meetings he attended, which were regularly held by some local organizations. He described the support group meetings with earnest:

These support group meetings are very helpful because, maybe four or five years ago, I wouldn’t have talked about this disease. I wouldn’t do anything. I kept to myself because I thought that my HIV was nobody’s business. But leaving all that stuff all bottled up inside, it just doesn’t help you. It hurts you in the long run, and prevents you from being at peace with it. So, the group meetings really helped get me there.

Lastly, many participants reported that they regularly utilized the services and programs of multiple community-based organizations in the Southern Nevada network to meet their health and practical needs, highlighting the explicit value of the strong connections among the many healthcare facilities, agencies, and organizations that belonged to the strong network of HIV-related services in their community. Burt (56 years old, Black, HIV-positive for 20 years), who utilized the HIV programs and services of four different organizations, pointed out that in addition to the great camaraderie of the providers within their community’s network of services, they have an official HIV awareness and health consortium in Southern Nevada composed of diverse providers from different clinics, community health centers, agencies, and organizations. According to Burt (56 years old, Black, HIV-positive for 20 years), the Southern Nevada HIV consortium has significantly supported the sense of community and multi-directional communication that has been engendered and enhanced by their community’s strong network of services.

#### 3.1.2. Emotional and Mental Health Support from Family of Origin or Chosen Family

A sense of community and the support group meetings held by the different community-based organizations were not the only dependable sources of emotional and mental health support that our participants brought up in their interviews. Participants discussed how vital it was for them to have the emotional and mental health support they received from their family of origin (i.e., parents, siblings, relatives, caregivers, and other people they grew up with) or their chosen family (i.e., peers, friends, partners, and other people they have purposefully chosen for mutual support and love) in order to promote their resilience. Our participants claimed that having staunch emotional and mental health support from their family of origin or chosen family helped them build their fortitude, which in turn, promoted their resilience to HIV/AIDS and its adverse clinical and social impacts.

Many participants expressed how their closeness to family members would help combat loneliness and give them a reason to keep fighting. Gar (61 years old, White, HIV-positive for 34 years) found his inner strength when he knew for certain that his family had his back. He narrated a story about a time when he leaned on his sister for help during a time of dire need:

When I found out about my HIV status, I eventually moved in with my sister and lived with her for a year. So that helped. I started to take my HIV medications and bounced back. Now I got 540 T-cells versus just two. So, my family has been instrumental in keeping me going, definitely. It’s almost as if, by just being there for me, they gave me a reason to live. When I was living alone, I was like, “Well, what am I doing this for?” I kind of gave up at a certain point, you know, like … the fight was getting to be too much.

Close family bonds brought greater meaning to the lives of many participants. Some participants expressed the importance of having family in their corner who supported them and cared for their well-being unconditionally. Robbie (68 years old, Black, HIV-positive for 11 years) shared his feelings of greater security and joy whenever his son visited him:

My son brings the grandkids by. He has a new baby that’s, like, one year old, and they come by, and they tear up the house. I’m glad when they leave, but I also love the fact that they’ve been here. It’s just a blessing to know that family is there and you have people that care about you, have your back, and look out for your interests. That’s always good to have.

Emotional and mental health support came from life partners as well. Some participants specifically described the benefits that came with having a partner who was also living with HIV/AIDS. Emotionally, being with someone who truly understood one’s own life circumstances and experiences brought a sense of safety, which considerably contributed to their resilience. For more practical reasons, it was beneficial to have someone who not only cared, but could also recognize the importance of keeping doctor’s appointments and taking HIV medications regularly. Charles (52 years old, Black, HIV-positive for 32 years) shared his own personal experiences:

My partner makes sure that I still take my meds because he takes his meds too. He takes his, and I take mine, and we kind of keep an eye on each other that way. He also tries to help others by being encouraging to a group of people that we co-mingle with. Yeah, he tries to keep up with the group, and keep them uplifted. So I try to keep him uplifted too.

In addition to partnerships, close friendships apparently served a similar role. Much like having a family of origin one can rely on for support, having the support of close friends who are part of one’s chosen family was often described by participants as a compelling reason to keep persevering. The struggles associated with living with HIV/AIDS can be draining to a person both physically and mentally, but having the care, concern, and understanding of close friends were key to promoting our participants’ resilience. According to Joseph (61 years old, White, HIV-positive for 38 years):

There was a dark period when, I thought I would be, you know, done. So I thought I should just get it over with, and I was ready to die. It was probably the limited close personal relationships that I did have that saved me. They made me think long and hard, and gave me hope.

It was evident that having the love and support of their chosen family or the family they grew up with was good reason for our participants to endure. Some participants explicitly mentioned their reasons for remaining or wanting to remain resilient, particularly to the clinical and social impacts of HIV/AIDS. For instance, Burt (56 years old, Black, HIV-positive for 20 years) elaborated on the hope he believed was inspired by his powerful familial connections, “I had regular motivating conversations with my friends, and there was always hope there. They encouraged me, and [because of that] I never thought I was going to die…I didn’t feel like HIV was going to kill me.”

### 3.2. Challenges to Promoting Resilience to HIV/AIDS

As our participants discussed factors that they believed facilitated the promotion of their resilience to HIV/AIDS, they also spoke about the challenges they experienced over the years that prevented them from promoting their resilience. Interestingly, each of these challenges were intrinsically related to the facilitators they described in their interviews.

#### 3.2.1. Absence of a Central Hub for HIV/AIDS Care and Services

While our participants lauded the fact that our region had a strong network of local HIV/AIDS prevention, treatment, and related social services, they were quick to point out the challenges related to having these numerous services and programs at separate clinics or agencies, particularly how these services and programs were so spread out across the region and difficult to access without having a dependable means of transportation. Our participants articulated that these significant challenges could potentially be addressed if there was a central hub that combined all the services and programs available in the region in one place and organized under one electronic records system.

Some participants who moved to Southern Nevada from other states in the last decade could not help but note the struggles they experienced commuting from one community-based organization to another to access the services and programs they needed, which they did not experience at the places they were previously residing. Gar (61 years old, White, HIV-positive for 34 years) described his own experiences trying to manage the services and programs he needed:

The only thing about Vegas is they don’t have one-stop shopping, so to speak. Like, I’m just used to it in Chicago, when you went to the doctor, and you could also see the therapist. You could get your labs done, see the doctor, and go to therapy, all in one visit. Those are all under one roof. Here, everything is so separate…and so far from each other. Sometimes, you even need to go to, like, five different places to get one thing done, so to speak. Now, I currently go to [a community health center]. Over there, they do the labs and social work as far as, um, case management is concerned. So that’s a good thing. That’s a plus. I hear they’re planning to set up a pharmacy there as well. It’s in the works. Things are getting a bit better. But when I first got here, it wasn’t as easy. It was like going to six places to get everything done.

This challenge was fundamentally linked to whether or not participants had a reliable means of getting around the region. Many participants relied on public transportation, and some did not always have the funds to cover the costs of public transportation. Jack (46 years old, Asian descent, HIV-positive for 23 years) explained how long one errand could possibly take to complete due to long bus routes, “It’s just really time consuming for those of us who don’t have a car and are taking the bus. To take the bus to do anything, it takes almost the entire day just to get one chore done.”

During certain times of the year, commuting around the region could be even more challenging. Robbie (68 years old, Black, HIV-positive for 11 years) shared one of his experiences traveling in the scorching heat of the desert in order to get to an HIV clinic, “In the summer months, it’s a little more difficult because, especially when you’re traveling by bus, it can be quite an ordeal just to be outside in the sun waiting all day for the bus to arrive.”

Our participants described other challenges they encountered in addition to the struggles of having to go through long journeys from one agency or clinic to another. The time commitment required to avail from certain services and programs, and the fact that some organizations were exceptionally busy, posed as additional challenges. For example, accessing grocery supplies from a very busy food bank may involve being placed on a long waiting list that could make a grocery trip become a whole day commitment. Peter (50 years old, Middle Eastern descent, HIV-positive for 29 years) described one of his experiences when he lined up for groceries:

There’s the food bank in [a city of Southern Nevada] where I used to go to and sit outside to get grocery supplies. We’d write down our name on a pad and go in the order we arrived. But you know, what I do is I go there at like three in the morning. And I just kind of like doze off in my car because I want to just get the supplies and leave. It’s my day off and I don’t want to spend my whole morning waiting in line. So I go the extra mile to get my groceries.

Aside from describing the difficulty of having to travel to several different places to access the services and programs they needed and making personal sacrifices to avail from such services and programs, our participants reported that they encountered challenges related to having to repeatedly complete forms and provide the same information at the different agencies since the agencies’ records were not necessarily always stored in one centralized system. Jay (41 years old, Black, HIV-positive for 12 years) expressed his frustration concerning this challenge:

I don’t know that it can necessarily be improved. I mean, Las Vegas offers a lot of resources, actually. The resources are amazing here! Sometimes, I guess the improvement would be maybe not to have to go through so many referrals and the same steps over and over from one place to another. There must be a way to make things [like meeting eligibility requirements] easier for everyone.

Despite the fact that having a strong network of local HIV/AIDS prevention, treatment, and related social services in our community meant that there was an abundance of resources to facilitate the promotion of our participants’ resilience to HIV/AIDS, the absence of a central hub that could provide all these resources’ services and programs under one roof apparently represented a substantial challenge that our participants still needed to overcome.

#### 3.2.2. Persistent HIV Stigma

The other challenge that we identified from our participant interviews is one that has long been pervasive since the start of the HIV/AIDS epidemic, HIV stigma. Participants discussed in their interviews aspects such as a lack of knowledge, ignorance, fear, and judgmental behavior, which have all been associated with HIV stigma, and linked to its obstinate persistence. Although many participants recognized that people from their family of origin or chosen family could be great sources of support, they recognized that family members could likewise potentially be culprits who help perpetuate the HIV stigma that poses as a huge challenge to their resilience-building. Many participants revealed that when they encountered HIV stigma, especially and often from close family members and friends, these experiences made them feel unsafe and reluctant about sharing their HIV status to others. Their feelings of uncertainty often added a complicated layer to their lives as they always felt that they constantly needed to carefully discern when and to whom they could disclose their HIV status. Despite having disclosed their status only to very few family members, Hugh (56 years old, Black, HIV-positive for 22 years) revealed that he still encountered stigma from those closest to him:

My brother still thinks if he uses the restroom after me that he might catch it. So he’s afraid to use the same toilet that I do. It’s that stigma…that stupid stigma. I don’t want to deal with it. Only a certain amount of people in my family actually know that I’m HIV positive but it’s still difficult.

Some participants shared how this history of prejudice, mistreatment, or even discrimination continued to affect them even though, in recent times, the social landscape has seemingly been changing. Although a lot of people may have become more knowledgeable and accepting of HIV, many of our participants who have been aging with HIV/AIDS over the last five to twenty years have spent the greater part of their lives hiding their HIV statuses to feel safe and have found it challenging to break this pattern of selective disclosure even to their loved ones. Tim (50 years old, Black, HIV-positive for 27 years) explained his continued reluctance to disclose his HIV status:

Um, socially, gosh, socially it’s been terrible. At first, there was no possibility of dating, no possibility of any intimacy. People are scared of you. Even at home, I was at my mom and dad’s house. And my dad was like, “Yeah you should get an electric razor for shaving because of all the little cuts. We don’t want blood on the towels if you cut yourself.” It definitely affected me for a while. And even though now, there’s a lot more of the U=U mentality…as long as you’re undetectable, you’re untransmissible, …people still find it a little hard mentally to wrap their heads around U=U. With such a long history of hiding, I’m still not one to share to others so readily.

The fear of being treated poorly extends beyond the boundaries of personal or familial relationships. Participants reported that many middle-aged and older MSM living with HIV/AIDS they knew feared being mistreated even in healthcare and larger social settings. Joseph (61 years old, White, HIV-positive for 38 years) knew someone living with HIV/AIDS who chose to access his care in a different city from where he lived and scheduled his clinic appointments very carefully out of fear of his HIV status being found out. Joseph recalled, “There was this one guy in one of my groups. He worked in entertainment. He was regularly driving from Las Vegas to [another city] to see his doctor because he didn’t want to be seen in a local doctor’s office!” According to our participants, the impacts of HIV stigma have continued to persist in the 21st century, and adversely affect them in their daily lives.

## 4. Discussion

Over recent decades, middle-aged and older MSM living with HIV/AIDS in Southern Nevada have encountered a variety of factors to promoting their resilience to HIV/AIDS, most of which appear to have existed in the context of their unique, sprawling urban landscape. As the largest urban area in Southern Nevada, Las Vegas is prospectively an important setting for CBPR on resilience to HIV/AIDS that is conspicuously distinct from other large urban areas in North America. Compared to other major cities (i.e., New York, Boston, Toronto) where quantitative research on resilience to HIV/AIDS has been previously conducted, Las Vegas is at least 100 years younger [39,40,41,42], and today, is still a fast-growing, rapidly urbanizing city with surging property development and population growth that has resulted in unparalleled lateral expansion to adjacent suburban and rural areas in the last two decades [43,44]. Las Vegas’ ongoing lateral expansion has led to a significant urban sprawl, which in turn, has led to a higher dependency on automobiles, longer commutes, greater greenhouse emissions, and much more remaining land for rededication to new construction and open space for infill development [45,46].

According to our participants, having a strong network of local HIV/AIDS prevention, treatment, and related social services in our community, as well as support from their family of origin or chosen family, have been crucial factors that served as facilitators to promoting their resilience. Our participants also revealed that the absence of a central hub for HIV/AIDS care and services in our community, as well as the persistence of HIV stigma in their daily lives, have been principal factors that proved to be challenges to their resilience-building. As we examined the positive and negative impacts of these different factors on the resilience-building of our participants, we began to recognize that these impacts have been mediated by the factors’ significant influence on different social determinants of health. From a social determinants of health perspective [36,37], factors that have a significant influence on critical determinants, such as access to quality health services, food security, housing stability, and social inclusion, could be more important than people’s individual health care or lifestyle choices in influencing their health [37].

Based on their interview narratives, the strong network of local HIV/AIDS prevention, treatment, and related social services that we have in our community had a significant positive influence on the social determinants of health that are relevant to our participants. These determinants include their ready access to quality health services, food security, and housing stability. In terms of their ready access to quality health services, the strong network of local HIV/AIDS prevention, treatment, and related social services in Southern Nevada significantly improved our participants’ access to HIV/AIDS care and services that not only have LGBTQI-affirming healthcare and service providers, but also providers who specifically have notable competency in caring for LGBTQI+ patients and clients. Research has documented that having LGBTQI-affirming providers, as well as providers who are highly competent in delivering care to LGBTQI+ patients and clients, are essential to providing quality care and services to MSM living with HIV/AIDS and other LGBTQI+ PLWH [49,50,51]. In terms of their food security, the strong network of services that we have in Southern Nevada has helped provide our participants with better access to food banks, affordable or free groceries, and supermarket vouchers and coupons. By addressing issues related to our participants’ food security, our community’s strong network of services has helped prevent problems associated with food insecurity, such as the exacerbation of hunger and medication side effects from the intake of HIV antiretroviral therapy [52,53], poor diet quality [54], non-adherence to the HIV antiretroviral therapy regimen [52], and risky sexual practices among MSM living with HIV/AIDS [55]. In terms of their housing stability, our community’s strong network of services has helped our participants gain better access to affordable housing opportunities, subsidized housing, supportive housing programs, and emergency shelters and transitional housing options. Studies have reported that unstable housing and homelessness are strikingly associated with poorer antiretroviral therapy and HIV program adherence among older MSM living with HIV/AIDS [56,57]. Thus, it has been critical and particularly helpful to our participants that they had been receiving invaluable housing support from our community’s strong network of local HIV/AIDS prevention, treatment, and related social services. Whether directly or indirectly, the significant positive influence of the strong network of local HIV/AIDS prevention, treatment, and related social services on the social determinants of health relevant to our participants has not only helped our participants improve their physical and mental health and overall wellbeing, but also facilitated the promotion of their resilience to HIV/AIDS.

Similarly, our participants pointed out that the emotional and mental health support they have received from their family of origin or chosen family has had a significant positive influence on their feelings of social inclusion. When our participants felt that they belonged and that they mattered to their families, were cared for, and valued, they were able to find firm and sustainable reasons to overcome their challenges related to living with HIV/AIDS, and persevere. Our participants reported that the help they had received from family and friends was paramount to their appreciation for life and willingness to maintain their health. Support, particularly from family members, friends, and partners, has been a well-documented aid to promoting resilience [20,58,59]. It has been recognized as a crucial facilitator of improved quality of life among older LGBTQI+ adults and PLWH [58,59].

Conversely, it was apparent from our participant interviews that the absence of a central hub for HIV/AIDS care and services in Southern Nevada has had a significant negative influence on various social determinants of health such as our participants’ ready access to quality health services, food security, and housing stability. Having a strong network of interconnected HIV clinics, community health centers, and community-based organizations, that have provided much-needed health and social services, has been incredibly valuable to middle-aged and older MSM living with HIV/AIDS in Southern Nevada, but our participants noted that these clinics, community health centers, and organizations must continue to make it a priority to be accessible so that their patients and clients could actually avail from their programs and assistance. While services such as public transportation and rideshares with free passes from community-based agencies have been available in recent years, there still seems to have been a large burden placed on PLWH. This is because the multiple locations they need to access for their care and services are widespread all across the Southern Nevada region. The inability of patients and clients to move around a large urban city could inadvertently lead to health complications, and the lack of access to affordable and reliable transportation has been a known issue for older PLWH for quite some time [60,61,62]. Historically, this major issue has been exacerbated in a sprawling city such as Las Vegas, where most of the health and social services for PLWH in the Southern Nevada region are located and spread out. These service locations make accessibility for PLWH difficult because they are not walkable distances and the temperatures are scorching in the peak of the summer, reaching as high as 120 °F [63].

When patients and clients have numerous visits to make to multiple healthcare and service providers in order to receive their HIV/AIDS care and services, practical challenges to accessing their providers, such as transportation issues, have been reported to negatively impact their adherence to their prescribed medications and clinic appointments [60]. Such transportation issues among middle-aged and older PLWH have been associated with worse health perceptions, pain, social functioning, health distress, and health transitions [61]. If transportation issues could be eliminated, or at the very least, significantly mitigated by establishing a central hub for all HIV/AIDS care and services in the community, middle-aged and older MSM living with HIV/AIDS and other PLWH would be able to not only access their HIV/AIDS care under one roof, but also avail from services that would help them achieve food security, housing stability, and even social inclusion. A central hub for all HIV/AIDS care and services would be able to help manage most, if not all, factors that influence social determinants of health, as well as ongoing engagement with patients and clients, which would be critical to devising novel interventions and strengthening existing programs aimed at improving outcomes across the HIV/AIDS care continuum [64].

Numerous research studies have suggested that social determinants of health such as access to quality health services, food security, housing stability, and social inclusion account for as much as 55% of health outcomes [37]. Related estimates have identified that the contribution of various factors in sectors outside of health to population health outcomes actually exceeds the contribution of factors from within the health sector itself. Addressing the social determinants of health appropriately is fundamental to improving population health and reducing longstanding inequities in health [37], including health disparities impacting MSM living with HIV/AIDS [18]. In the specific context of our participants residing and obtaining HIV-related healthcare and social services in Las Vegas and Southern Nevada, interventions that could be utilized to address the relevant social determinants of health could potentially benefit from considering policies and programs that would reverse decades of ongoing sprawl. For example, smart, land-efficient policies, zoning regulations, and public infrastructure investments, which favor high-density and mixed-use infill land development that prioritizes affordable housing and other essential services, accessible and well-designed public transit options, the reduction of automobile dependence, and the promotion of walkable communities could prospectively help address the social determinants of health that are relevant to our participants [44,45,46].

Finally, our participants shared that the persistence of HIV stigma in their lives has, likewise, had a significant negative influence on their feelings of social inclusion and non-discrimination. Previous research has frequently discussed the ongoing detrimental effects of stigma, and the higher rates at which PLWH encounter it [65,66]. Despite the fact that we only required a minimum of one year of experience of living with HIV/AIDS in our inclusion criteria, all of our participants reported that they had lived with HIV/AIDS for more than 10 years, save for one participant who had been living with HIV/AIDS for seven years at the time of their interview. Over 80% of our participants had been living with HIV/AIDS for more than 20 years at the time they participated in our study, and there were no significant details that stood out in the interview responses of the three participants who had been HIV-positive for less than two decades in terms of their perspectives and lived experiences related to living with HIV/AIDS and their resilience-building. Among our 16 participants, only one reported that they were diagnosed with HIV/AIDS a couple of years before the advent of clinically approved therapy for the condition. This meant that the particular focus of the findings of our study was explicitly based on the responses of middle-aged and older MSM who not only have substantial experiences living with HIV/AIDS based on the number of years they had been HIV-positive, but who also have considerable experiences related to the availability of and continuous updates in HIV/AIDS treatments, and just as importantly, the changes associated with HIV stigma during their life course.

Meyer has discussed common experiences of people affected by stigma such as expectations of rejection, hiding and concealing their identities, and reluctance to self-disclose to significant others [65], experiences that were described by many of our participants. Although some of our participants shared that their encounters with HIV stigma have not been as often as they were in earlier decades, they recognized that they still automatically resorted to old habits to protect themselves in certain circumstances or situations when they could reasonably expect the possibility of encountering discrimination and social exclusion from stigma. This recurring hypervigilance for stigma, in turn, has made it harder for them to freely connect with others and has remained an insidious challenge to their resilience-building. Present in both social and institutional settings, the effects of stigma can often feel inescapable, particularly for older adults living with HIV/AIDS who have experienced these effects for most of their life [67]. The documented detrimental effects of stigma on PLWH have included higher rates of depression, lower rates of social support, and poorer quality of life [68,69,70].

The findings of our study add important knowledge to the growing body of the academic literature focused on examining factors that promote the resilience of middle-aged and older MSM living with HIV/AIDS. Based on the input and feedback of our participants, our study identified critical factors that have a strong influence on known social determinants of health, which are crucial to mediating the identified factors’ impacts on our participants’ efforts to promote their resilience to HIV/AIDS, especially in the context of residing in Southern Nevada.

### Limitations of the Study

As we recognize the contributions of our study to the extant literature, it is important for us to acknowledge the limitations of our study. One limitation of our study is related to its participant recruitment strategy. Since our participants were primarily recruited with the help of our Southern Nevada partner clinics, community health centers, and community-based organizations, it stands to reason that many of our participants would be active service users of these clinics, health centers, and organizations, and would likely have a healthy appreciation for the care and services they provide. It was unlikely that we were able to access much of the perspectives and lived experiences of middle-aged and older MSM living with HIV/AIDS from Southern Nevada who did not avail from the services of our community partners.

Another limitation of our study is the limited diversity of our participants. At the beginning of our study, it was our intention to recruit participants with a wide range of experiences living with HIV/AIDS in terms of the number of years they had been HIV-positive. As it turned out, over 80% of the participants we recruited were long-term survivors who had been living with HIV/AIDS for over 20 years. Thus, we were unable to examine the experiences of middle-aged and older MSM who had been recently diagnosed and living with HIV/AIDS for less than five years. We suspect that this outcome may be due to the possibility that most MSM living with HIV/AIDS from our community who were eligible and willing to participate in our study were also those who have had more experiences and confidence in joining research studies over the last two decades. Nonetheless, this meant that our study was unable to draw the range of participant experiences we hoped we would gather. Related to this, although we received substantial support from our community partners to recruit a diverse range of participants from Southern Nevada in terms of their ethnoracial background and how they identified in terms of their sexual identity (e.g., gay, bisexual, queer, MSM), only 50% of the participants we were able to recruit identified as ethnoracial minorities, and they all identified as gay. We acknowledge the significant importance of other critical factors to the resilience-building of middle-aged and older MSM living with HIV/AIDS, which we were unable to discuss in this article as they were factors cognate to but outside of the WHO social determinants of health perspective, and not within the scope of our study. Despite this, we recognize that critical factors such as race, ethnicity, and other sociodemographic factors are crucial influences to the promotion of the resilience of middle-aged and older MSM to HIV/AIDS based on the findings of prior related research [30,31]. Future research would likely be able to gain even more diverse perspectives and lived experiences, and consequently more knowledge, if they are able to include participants through a wider range of recruitment strategies, as well as involve more participants who identify as ethnoracial minorities, as well as bisexual, queer, or simply as MSM.

Lastly, it is important to emphasize that the findings of our study are restricted to the context of the Southern Nevada region, and are potentially applicable only to other relatively younger, fast-growing, and sprawling urban regions of North America that have an increasing number of HIV/AIDS services spread across the widening extent of their landscape. Despite the limitations we have enumerated, our study was able to address several research gaps by not only meaningfully engaging relevant community stakeholders from our region in order to identify and describe our stakeholders’ own definition of their resilience to HIV/AIDS and the factors that they believe promote it, but also examining our qualitative findings based on the rich perspectives and lived experiences of middle-aged and older MSM living with HIV/AIDS in Southern Nevada from a distinct social determinants of health perspective [37].

## 5. Conclusions

The results of our study corroborate the findings of previous research while presenting discoveries and lessons specific to the experiences of middle-aged and older MSM living with HIV/AIDS in Southern Nevada, which may prove useful for future research in urban locations with sprawling and other similar characteristics. Our study identified the importance of critical factors such as a strong network of local HIV/AIDS prevention, treatment, and related social services; emotional and mental health support from family of origin or chosen family; a central hub for all HIV/AIDS care and services in the community; the elimination of HIV stigma; and the mediating power of relevant social determinants of health (i.e., access to quality health services, food security, housing stability, and social inclusion) to promote the resilience of middle-aged and older MSM living with HIV/AIDS. To date, as far as we can determine, only a few empirical studies have taken advantage of the social determinants of health perspective in the conduct of their research to examine the resilience of MSM living with HIV/AIDS and other PLWH [35,71,72]. Future studies focused on the promotion of resilience to HIV/AIDS could benefit from conducting research utilizing a social determinants of health perspective, particularly to identify other facilitators, challenges, and factors related to the resilience-building of middle-aged and older MSM living and aging with HIV/AIDS in the 21st century.

## Figures and Tables

**Table 1:** Participant Demographics (*n* = 16).

Pseudonyms	Age	Identified as	Race	Years HIV+
Roy	41	Gay	White	7
Jay	41	Gay	Black	12
Gar	61	Gay	White	34
Peter	50	Gay	Middle Eastern	29
Kris	45	Gay	White	23
Robbie	68	Gay	Black	11
Hugh	56	Gay	Black	22
Mark	58	Gay	White	23
Joseph	61	Gay	White	38
Charles	52	Gay	Black	32
Burt	56	Gay	Black	20
David	61	Gay	White	28
Jack	46	Gay	Asian	23
Jimmy	64	Gay	White	42
Bart	54	Gay	White	26
Tim	50	Gay	Black	27

## Data Availability

The data presented in this study are available on request from the corresponding author. The data are not publicly available due to privacy and ethical reasons.
